# T-type channels in neuropathic pain - Villain or victim?

**DOI:** 10.1080/19336950.2020.1740487

**Published:** 2020-03-13

**Authors:** Norbert Weiss

**Affiliations:** Institute of Organic Chemistry and Biochemistry, Czech Academy of Sciences, Prague, Czech Republic

**Keywords:** Pain, calcium channel, T-type channel, post-translational modifications, ubiquitinylation, phosphorylation, glycosylation, sumoylation

Neuropathic pain syndromes affect between 30 and 50% of the world population and represent a significant burden for patients, society, and healthcare systems. Many hypotheses have been formulated about the mechanisms of neuropathic pain among which elevated expression of T-type calcium channels in peripheral nociceptive nerve fibers (so-called “nociceptors”) is seen as a hallmark in several experimental pain models [1]. Nociceptors have their cell bodies in the dorsal root ganglia (DRG) and express predominantly the Ca_v_3.2 channel subtype whose primary function is to regulate neuronal firing and synaptic transmission at dorsal horn synapses [2]. Given these important functions in peripheral sensory neurons, aberrant expression of T-type channels in primary pain fibers comes as a pertinent cellular mechanism of neuropathic pain syndromes. How this up-regulation of T-type channels occurs at a mechanistic level has been the subject of a great deal of research in recent years and several studies pointed to a role of post-translational modification of the channel protein.

**Figure 1.**
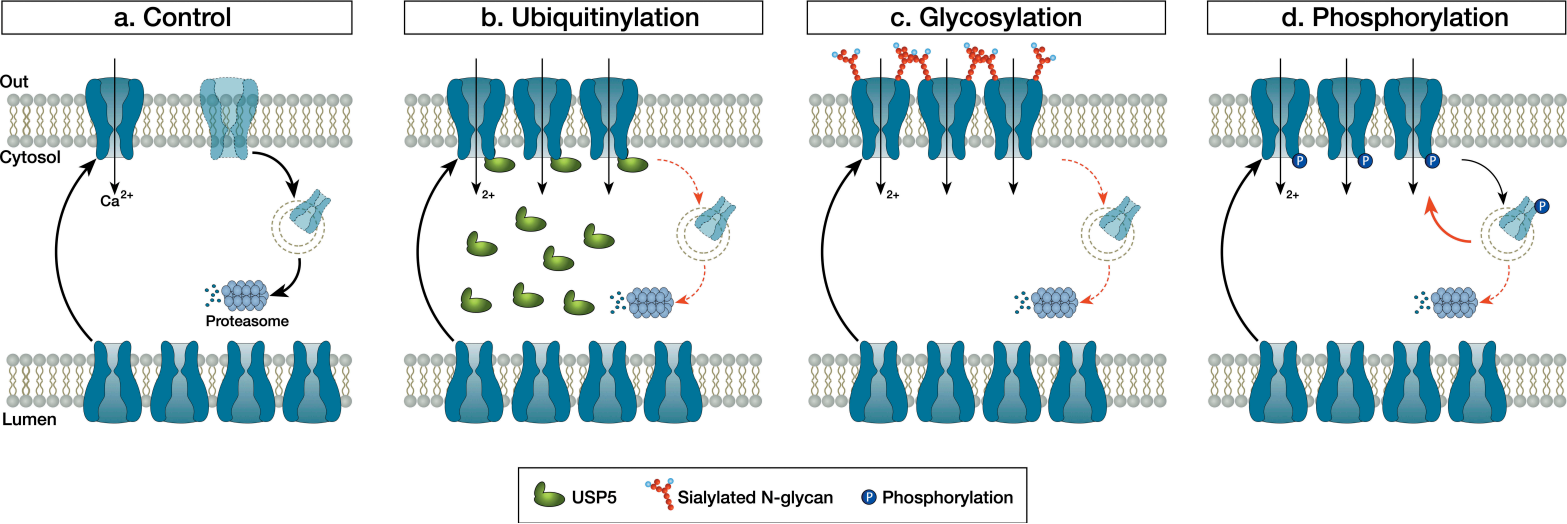
Summary of the post-translational modifications implicated in the up-regulation of Ca_v_3.2 during neuropathic pain conditions. Under normal conditions, surface expression of Ca_v_3.2 is balanced by the number of channels coming to and being removed from the cell surface. Decreased ubiquitinylation of Ca_v_3.2 following the up-regulation of the deubiquitinylating enzyme USP5 diverts Ca_v_3.2 from proteasomal degradation (b). Similarly, increased glycosylation of Ca_v_3.2 and/or modification of the glycan structure stabilizes the channel in the plasma membrane (c). Finally, increased phosphorylation of Ca_v_3.2 potentiates the recycling of the channel back to the plasma membrane (d).

Post-translational modification refers to changes a protein may undergo after translation (cleavage and/or covalent addition of chemical moieties) and serves as a secondary level of control to fine tune its functional expression. While post-translational modification of proteins is an essential part of cellular homeostasis, it has become increasingly evident that this process is altered in pathological conditions including pain syndromes. Using a mouse model or peripheral nerve injury-induced neuropathic pain, Garcia-Caballero et al., reported a decreased ubiquitinylation of Ca_v_3.2 channels in primary afferent nerve fibers [3]. Biochemical analysis revealed that this effect was mediated by the up-regulation of the deubiquitinylating enzyme USP5 resulting in the accumulation of Ca_v_3.2 in the plasma membrane. Importantly, the authors showed that prophylactic knockdown of USP5, or prophylactic disruption of the Ca_v_3.2/USP5 complex, was sufficient to prevent nerve-injury-induced mechanical and thermal hyperalgesia demonstrating the causal implication of the ubiquitinylation machinery in the development of neuropathic pain in this experimental model. In yet another study using the same experimental pain model, the authors reported a decreased SUMOylation of USP5 in peripheral nociceptive nerve fibers [4]. Given that SUMOylation of USP5 negatively regulates its ability to interact with Ca_v_3.2, decreased SUMOylated USP5 during nerve injury would favor Ca_v_3.2/USP5 interaction. This would add to the already elevated level of USP5, which would enhance the deubiquitinylation of Ca_v_3.2 and further potentiate the expression of the channel in the plasma membrane.

Asparagine (N)-linked glycosylation is another type of post-translational modification that has been reported to potentially contribute to peripheral painful diabetic neuropathy. Several *in vitro* studies have documented the functional importance of N-glycosylation for Ca_v_3.2 channels [5–7]. Of particular relevance for painful diabetic neuropathy is the observation that surface expression of recombinant Ca_v_3.2 is enhanced in cells exposed to elevated glucose levels and this phenomenon requires the glycosylation of the channel at asparagines N192 and N1466. Along these lines, Orestes et al., demonstrated that *in vitro* removal of sialic acid moieties from complex glycan structures using neuraminidase normalized T-type currents in DRG neurons from *ob*/*ob* mice (a transgenic model of peripheral painful diabetic neuropathy), and produced analgesia *in vivo* [8]. The observation that neuraminidase had no effect on T-type currents in wild-type neurons would suggest an elevated degree of sialyzation during diabetes, which could have contributed to the up-regulation of Ca_v_3.2 channels. At this stage, it is not clear whether this effect relies on the sialyzation of Ca_v_3.2 per se, or on the sialyzation of other cellular components that could have influenced the expression of the channel. Furthermore, whether this alteration is due to increased activity of sialyltransferases or decreased activity of neuraminidases remains to be established.

Finally, two recent studies pointed to a role of T-type channel phosphorylation in the development of chronic pain syndromes. Our group reported that homocysteine, an intermediate product in the conversion of methionine to cysteine, enhances surface expression of Ca_v_3.2 and contributes to the development of mechanical allodynia in a rodent model of homocysteinemia. This mechanism was attributed to the phosphorylation of Ca_v_3.2 by PKC at serines S532, S1144, and S2188 and resulted in an enhanced recycling of the channel back to the plasma membrane. Another study reported that Cdk5-dependent phosphorylation of Ca_v_3.2 at serines S561 and S1987 also contributes to the expression of the channel in the plasma [9]. Importantly, these authors found that Cdk5 is up-regulated following spinal nerve injury and pharmacological inhibition of Cdk5 reversed mechanical allodynia.

In conclusion, post-translational modifications potently modulate the expression of T-type channels (Figure 1) and there is increasing evidence that these regulations could have a primary role in the pathological switch from nociceptive to chronic pain conditions. Pharmacological studies have demonstrated the analgesic potential of pan T-type channel blockers in animal models of neuropathic pain and early clinical studies [10]. However, these molecules usually lack selectivity and do not discriminate between physiological and pathological pain. Therefore, targeting the regulatory pathways involved in the pathological switch of T-type channels could potentially allow for a selective modulation of chronic pain syndromes without altering their physiological functions.
